# A patient with Turner syndrome received the percutaneous vertebroplasty seven times: a case report and literature review

**DOI:** 10.1186/s40001-021-00617-4

**Published:** 2021-12-07

**Authors:** Longyu Li, Yifang Shi, Nan Zhao, Zhengpei Liu, Zhe Zhao, Zongmian Song, Sailei Zheng, Miaoheng Yan, Zikuan Leng, Songfeng Chen, Guowei Shang, Hongwei Kou, Hongjian Liu

**Affiliations:** Department of Orthopaedics, The First Affiliated Hospital of Zhengzhou University, No. 1 Jianshe East Road, Zhengzhou, China

**Keywords:** Osteoporosis, Turner syndrome, Percutaneous vertebroplasty, Vertebral compression fractures, Estrogen, Gut microbiome

## Abstract

**Background:**

Turner syndrome (TS) is characterized as the complete or partial absence of one X chromosome and is an extremely rare disease affecting approximately 1:2500 live female births. Though the prevalence of osteoporosis among women with TS is estimated to be around 55–64% and they suffer more frequently from fractures than normal, few reports concerning TS patients with osteoporosis are able to be seen due to tiny number of patients.

**Case presentation:**

Here, we report a rare case of TS with osteoporosis, who has undergone percutaneous vertebroplasty (PVP) seven times because of several vertebral compression fractures (VCFs). G-banded karyotype analysis was performed and the result was 45,X[43]/47,XXX[17], indicating that the patient was a mosaicism of TS karyotype and Trisomy X syndrome karyotype. TS is the underlying cause of low level of estrogen for this patient. The interaction of aging, estrogen deficiency and intestinal dysbacteriosis leads to her severe osteoporosis and multi-segmental VCFs. The aim of this report is to provide recommendations regarding the management of TS patients with osteoporosis by reviewing the clinical presentation of TS, the influence of estrogen deficiency in osteoporosis, etc.

**Conclusions:**

Early diagnosis and hormone replacement treatment are essential for TS patients to prevent osteoporosis and reduce the risk of fractures. This is a rare case report describing TS patient with severe osteoporosis and VCFs.

## Introduction

Turner syndrome (TS) is characterized as the complete or partial absence of one X chromosome and is an extremely rare disease affecting approximately 1:2500 live female births. The most common karyotype causing TS is 45,X, found in 45% of live births. The incidence of 45,X/47,XXX mosaicism among patients with TS is less than 5% [1]. It is estimated that the incidence of osteoporosis among TS patients is about 55–64%. Women with TS have a higher fracture risk than healthy individuals because there is a cortical density reduction among these patients [2].

Herein, we report the case of a patient who was diagnosed as severe osteoporosis with TS and received the percutaneous vertebroplasty seven times due to vertebral compression fractures (VCFs).

## Case presentation

Our patient was a 65-year-old woman who was enrolled in the orthopaedics department of this hospital because of lumbar and back pain after seven times of percutaneous vertebroplasty (PVP).

The patient had been healthy until September 2019, when she got a fall carelessly and suffered from severe low back pain. She was evaluated in a local hospital because the pain was not alleviated after a rest. The lumbar spine X-ray showed the VCFs at L1 and L4. She underwent the first PVP to stabilize the fractured vertebral bodies and relief the symptom.

The patient had a severe backache again because of falling down from the bed in July 2020. According to the X-ray, the patient underwent the PVP for the second time due to a new VCF at T11. One month later, she felt low back pain again after bending forward to pick up a potted flower. X-ray showed that there was an obvious VCF at T9 and the patient had another PVP. Though the lumbar and back pain has been relieved after three times surgeries, she took the 600 mg calcium and 125 IU vitamin D daily since then.

In September 2020, the patient felt lumbar pain again without any recognizable precipitating factors. Bone mineral density (BMD) was measured with ultrasound BMD analyzer and the speed of sound (SOS) was 4016 m/s, indicating that T-score of the patient was − 1.5. VCF at L2 could be seen clearly by X-ray and magnetic resonance imaging (MRI). Owing to this, she underwent a PVP for L2 immediately. After 2 months the low back pain became worse without any clear reasons. A new VCF at L3 was found and another PVP for L3 was administered. Additionally, the patient underwent another PVP at T12 10 days later because of the aggravation of pain after bending from the waist. Though the situation of the patient was not improved this time and bone cement leakage occurred, no neurologic complications happened to her fortunately.

Apart from that, her general past medical history was remarkable. The patient had amenorrhea all the time, which means menarche had not happened ever and the lifelong absence of menses.

The patient underwent cholecystectomy 10 years ago because of gallbladder stones. Chronic superficial gastritis also confused her for almost 8 years and she had the resection of gastric polyp in January 2021. Coronary heart disease (CHD) was diagnosed 5 years ago and stent was implanted in the stenosed artery. Doctors decided to implant another scaffold for the exacerbation of chest pain in January 2021. However, the operation was canceled after assessing and concluding that the patient could not bear the surgery due to her poor health.

**Fig. 1:**
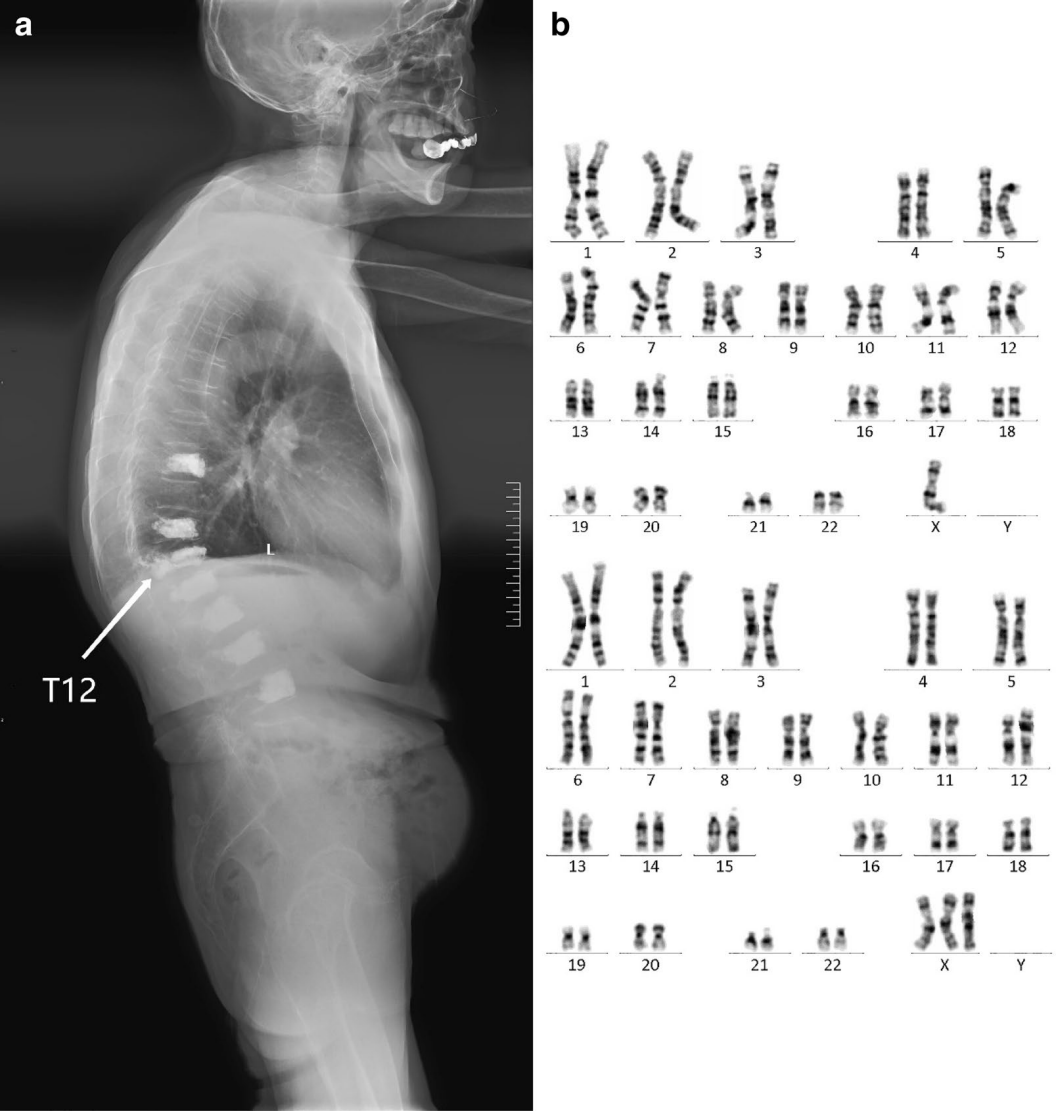
Imaging studies and karyotype analysis. **a** The whole spine erect lateral projection radiograph shows the patient had received seven times of PVPs at T9, T11–L4. The minimum scale of the ruler in this radiograph represents 5 mm. **b** The result of G-banded karyotype analysis shows the karyotype of this patient is 45,X[43]/47,XXX [17]

The patient was transferred to this hospital for further evaluation and treatment after seven PVPs. BMD was measured again with dual-energy X-ray absorptiometry (DEXA) and the T-score was − 4.1. The whole spine erect lateral projection radiograph displayed seven vertebral bodies injected bone cement clearly (Fig. 1a).

Physical examination for this patient demonstrated short stature, short neck, and no breast development. Since amenorrhea is one of the most significant clinical manifestations of this patient, a three-dimensional transvaginal ultrasound examination was considered for differential diagnosis. The result indicated suspected congenital infantile uterus and the structure of bilateral ovaries was not observed. The levels of six serum sex hormones were measured and the result suggested low levels of estrogen and testosterone (Table 1). However, other etiologies could not be distinguished by the examinations that already had done for the reason that aging may affect all these results, especially the levels of sex hormone. Owing to this, G-banded karyotype analysis was performed and the result was 45,X[43]/47,XXX[17], indicating that the patient was a mosaicism of TS karyotype and Trisomy X syndrome karyotype (Fig. 1b).

**Table 1:** Laboratory results

Variable	Evaluation at our center	Reference range
Six serum sex hormones^a^		
Follicle stimulating hormone (mIU/mL)	56.54	25.80–134.80
Luteinizing hormone (mIU/mL)	23.79	7.70–58.50
Prolactin (ng/mL)	12.69	4.79–23.30
Estrogen (pg/mL)	5.00	5.00–138.00
Progesterone (ng/mL)	0.09	< 0.05–0.126
Testosterone (ng/mL)	0.025	0.029–0.408
Parathormone (pg/mL)	31.70	15.00–65.00
25-Hydroxyvitamin D (ng/mL)	27.59	> 18.00
Serum calcium (mmol/L)	2.23	2.00–2.70
Serum phosphorus (mmol/L)	1.42	0.81–1.90
Alkaline phosphatase (U/L)	62	35–105

^a^The ranges of six serum sex hormones are for postmenopausal women. They may therefore not be appropriate for all patients

## Discussion

G-banded karyotype analyses showed that the karyotype of this patient was 45,X[43]/47,XXX[17], meaning that 60 cells were randomly selected totally, and 43 cells and 17 cells displayed a 45,X karyotype and 47,XXX karyotype, respectively. This result indicated that the patient had a mosaicism and Turner karyotype was in the overwhelming majority. Previous reports have demonstrated that 45,X/47,XXX mosaicism has a milder phenotype than 45,X karyotype. This mosaicism has a lower likelihood of cardiovascular and skeletal anomalies compared with common TS patients [3]. The presence of XXX cells makes it more likely to retain the ovarian function and fertility [4]. Unfortunately, though the patient in this case was a typical 45,X/47,XXX mosaicism, she still suffered from primary amenorrhea, cardiac disease and severe osteoporosis for the reason that cells which had 47,XXX karyotype were in a decided minority. Because 45,X/47,XXX karyotype is rare, more studies are needed to refine the analysis of the genotype–phenotype associations. A more in-depth description of the link of different karyotypes to osteoporosis and fragility fractures would be a focus for future research.

It has been confirmed that it is hormonal imbalance and intrinsic bone abnormalities together that result in the increase of skeletal fragility in women with TS [2]. According to the result of ultrasound examination, it was highly likely that the patient had no ovaries and her uterus was in the primitive state combined with clinical history. This patient had decreased level of estrogen and testosterone compared with normal references. Estrogen deficiency plays an important role in the pathophysiology of osteoporosis. The relevant mechanisms of estrogen include affecting bone metabolism via releasing the bone-active cytokines, stimulating the proliferation and differentiation of regulatory T (T-reg) cells to be anti-osteoclastogenic, etc. [5, 6]. In addition, follicle stimulating hormone (FSH) also participates in the process of bone metabolism, which is able to affect the formation and activity of osteoclasts by binding to the FSH receptor expressed on osteoclasts or enhancing the production of the pro-osteoclastogenic cytokine tumor necrosis factor-α (TNF-α). High FSH serum level may be detected in the prepubertal period of patients with TS [7]. But for the patient, this could not be observed due to aging.

It is reported that women with TS have a higher risk of fracture compared to normal even with normal T score [2]. T score of the patient was − 4.1, indicating that she suffered from severe osteoporosis. The SHOX gene, which is located at the pseudoautosomal region of sex chromosomes, is known as one of the major causes of intrinsic bone abnormalities. SHOX deficiency leads to short stature, short neck and bone geometry changes, in particular for cortical density reduction, which is a common feature of bone fragility in TS [7]. It is noted that typical short stature itself cannot predispose TS patients to fractures.

It is the abnormality in spine and pelvic parameters that resulted in the lack of ideal spinal balance for this patient. Thoracolumbar kyphosis (42°) and pelvic tilt (30°) of the patient were larger than those of normal after measuring, which may cause the biomechanical disadvantage, affect the spinal stability and increase the risk of fractures. Besides, cluster phenomenon (at least five thoracic or lumbar VCFs within a period of 8 months) after initial PVP also affected the force situation of spine [8].

Digestive diseases, such as inflammatory bowel disease and so on, are typical comorbidities for TS patients. Chronic inflammation owing to gastritis may affect bone metabolism by reducing the absorption of calcium and multiple micronutrients. On the other hand, chronic superficial gastritis of the patient influenced her gut microbiome. Fecal samples were collected and undergone the gut microbiota test, indicating that the kinds of beneficial bacteria was rare though the diversity of gut microbiota kept at a high level (340 kinds of bacteria were found). Besides, several kinds of bacteria which had the ability to produce short-chain fatty acids (SCFAs) accounted for less than 8% of all tested bacteria. Researches have confirmed that gut microbiome is relevant to osteoporosis extremely [9]. SCFAs produced by intestinal flora through fermenting dietary fibers have the ability to inhibit bone resorption without affecting bone formation [10]. Additionally, intestinal microbes can regulate the differentiation and apoptosis of osteoclast by controlling the dynamic balance of Th-17/T-reg cell [11, 12].

Aging of the patient was another cause of her severe osteoporosis. Only the level of estrogen being considered is not comprehensive. It is believed that the substantial majority of trabecular bone loss during the life is age-related and estrogen-independent [13]. Increased oxidative stress (OS) caused by advancing age is a fundamental mechanism of the loss of bone mass and strength. It is excessive accumulation of reactive oxygen species (ROS) that leads to age-related changes including osteopenia. However, a close association is also recognized between aging and estrogen. Estrogen deficiency decreases the defence of body against OS and may aggravate adverse effects of aging on both bone and lipid metabolism [14]. This is able to explain why the patient suffered from both osteoporosis and CHD to some extent.

Early diagnosis of TS is important and sex chromosome abnormalities can be detected by prenatal diagnosis, such as chorionic villous sampling and amniocentesis. Karyotype analysis is the gold-standard technique for diagnosing TS currently. Newborn screening of TS, which is the new technique, has been proposed recently including pyrosequencing and whole-exome sequencing [15].

Owing to the wrong reduction of T score for patients with TS, the prevention of osteoporosis and high fracture risk is essential even the T score is at normal range. Vitamin D deficiency is recommended to screen for girls with TS at the age of 9 to 11 and the concentration of serum vitamin D is monitored every 2–3 years [2].

Bone turnover markers (BTMs), including N-terminal propeptide of type I procollagen (PINP), β-cross-linked C-telopeptide of type I collagen (β-CTX) and molecular fragment of osteocalcin N-terminal (N-MID), can evaluate bone resorption and formation. The sensitivity of BTMs is higher than BMD measured by DXEA to assess the effect of anti-osteoporosis therapy and predict the risk of refractures [16]. Therefore, it is appropriate to monitor BTMs more frequently than BMD, especially for TS patients. Monitoring BTMs every 3 months and BMD every 6 months or 1 year is valid if possible.

Hormone replacement therapy used in the treatment of patients with TS at younger ages may provide benefit for bone quality and decreased fragility fractures in the future [17]. Considering the age of this patient, no particular treatment for TS is needed for the reason that she had no demand and indeed for fertility. Estrogen replacement therapy is not the key point of her management. However, calcium and vitamin D supplement is necessary and monitoring them every 3 months is suitable for the patient.

Surgical treatment for VCFs is restricted to patients with neurological deficit because of compression of the spinal cord or the cauda equina normally. Sometimes patients with severe spinal deformity after fractures are also able to be considered to undergo operative treatment. Although surgical treatment can relieve neural compression and restore the spinal sequence, it is not suitable for patients with osteoporosis. Osteoporosis is the relative contradiction of surgery and low bone mass may lead to unstable pedicle screws fixation. Surgical treatment was associated with great operative difficulty and high risk for this aging patient with CHD. Besides, the visual analogue scale (VAS) score of the patient was greater than or equal to 6. Considering the combination effect of spinal imbalance, TS and osteoporosis, PVP was better than operation for the reason that great possibility of screws loosening might lead to poor prognosis for this patient.

PVP has been widely used around the world for treating painful VCFs during the last two decades. It is believed as a safe and effective technique to release pain and improve functional status [18]. Although some authors declare that the incidence of new fractures in adjacent vertebrae may increase for the patients who were treated by PVP previously, it seems that it is osteoporosis rather that PVP that leads to new adjacent VCFs [19]. The latest MRI suggested a suspected new fracture at T12. However, the bone cement had been injected in its vertebral body. Considering the patient had undergone PVP seven times, anti-osteoporosis therapy rather than another PVP is the most important and appropriate management for this patient next phase.

Recently researches have confirmed that pedicle screw fixation combined with PVP in the treatment of VCFs is superior to PVP alone to restore vertebral height and relieve pain [20]. This may be a new idea for the management of TS patients with osteoporosis who cannot receive hormone replacement therapy in the future.

## Conclusions

TS is the underlying cause of low level of estrogen in this patient. The interaction of aging, estrogen deficiency and intestinal dysbacteriosis had lead to her severe osteoporosis and multi-segmental VCFs. Hormone replacement therapy is not suitable due to aging. Anti-osteoporosis therapy is the most appropriate management for her in the long run. For patients with TS, early diagnosis and early hormone replacement treatment are essential to prevent osteoporosis and reduce the risk of fractures.

## Data Availability

The datasets used and/or analyzed during the current study are available from the corresponding author on reasonable request.

## Abbreviations

TS: Turner syndrome
PVP: Percutaneous vertebroplasty
VCF: Vertebral compression fracture
BMD: Bone mineral density
SOS: Speed of sound
MRI: Magnetic resonance imaging
CHD: Coronary heart disease
DEXA: Dual-energy X-ray absorptiometry
T-reg: Regulatory T
FSH: Follicle stimulating hormone
TNF-α: Tumor necrosis factor-α
SCFAs: Short-chain fatty acids
OS: Increased oxidative stress
ROS: Reactive oxygen species
BTM: Bone turnover marker
PINP: N-terminal propeptide of type I procollagen
β-CTX: β-Cross-linked C-telopeptide of type I collagen
N-MID: Molecular fragment of osteocalcin N-terminal
VAS: Visual analogue scale
